# Applied Investigation of Methyl, Ethyl, Propyl, and Butyl Mercaptan as Potential Poisons in the Gas Phase Polymerization Reaction of Propylene

**DOI:** 10.3390/polym16202851

**Published:** 2024-10-10

**Authors:** Joaquin Hernandez-Fernandez, Juan Esteban Herrera Zabala, Edgar Marquez

**Affiliations:** 1 Chemistry Program, Department of Natural and Exact Sciences, San Pablo Campus, University of Cartagena, Cartagena 131001, Colombia; 2 Chemical Engineering Program, School of Engineering, Universidad Tecnologica de Bolivar, Parque Industrial y Tecnológico Carlos Vélez Pombo, Km 1 Vía Turbaco, Turbaco 130001, Colombia; 3 Department of Natural and Exact Science, Universidad de la Costa, Barranquilla 080002, Colombia; 4 Grupo de Investigación en Ciencias e Ingeniería, Chemistry Program, Department of Natural and Exact Sciences, San Pablo Campus, University of Cartagena, CECOPAT&A, Cartagena 131001, Colombia; jherreraz@unicartagena.edu.co; 5 Grupo de Investigaciones en Química y Biología, Departamento de Química Y Biología, Facultad de CienciasBásicas, Universidad del Norte, Carrera 51B, Km 5, Vía Puerto Colombia, Barranquilla 081007, Colombia; ebrazon@uninorte.edu.co

**Keywords:** mercaptans, polypropylene, catalytic productivity, Ziegler–Natta catalyst, inhibitors, trace level impurities, polymerization

## Abstract

The polypropylene (PP) synthesis process is crucial in the plastics industry, requiring precise control as it directly impacts the catalytic activity and the final product’s performance. This study investigates the effects of trace amounts of four different mercaptans on the polymerization of propylene using a fourth-generation Ziegler–Natta (ZN) catalyst. Various concentrations of these mercaptans were tested, and results showed that their presence significantly reduced the melt flow index (MFI) of the final PP. The most notable MFI decrease occurred at 37.17 ppm of propyl mercaptan and 52.60 ppm of butyl mercaptan. Methyl and ethyl mercaptan also reduced the MFI at lower concentrations, indicating that mercaptans act as inhibitors by slowing down the polymerization process and reducing the fluidity of molten PP. The highest MFI increase was observed at lower concentrations of each mercaptan, suggesting that smaller molecular inhibitors require less concentration. This trend was also seen in the catalyst’s productivity, where lower concentrations of methyl mercaptan reduced PP production more effectively than higher concentrations of butyl mercaptan. Fourier transform infrared spectroscopy (FTIR) identified interactions between the mercaptans and the ZN catalyst. Computational analysis further supported these findings, providing insights into the molecular interactions and suggesting possible inhibition mechanisms that could impact the final properties of polypropylene.

## 1. Introduction

The fourth-generation Ziegler–Natta catalyst plays a fundamental role in the industrial manufacture of polyolefins, especially iso-tactic polyethylene and polypropylene, being recognized as one of the most prominent catalysts in this process [1,2,3,4]. In 1953, Karl Ziegler made a crucial discovery when he observed that certain transition metal compounds, such as titanium, vanadium, and zirconium, combined with aluminum alkyls, could catalyze the polymerization of alkenes. This discovery was significant and promising, given that this process could be carried out at temperatures and pressures lower than those required in radical polymerization [5,6]. The following year, Giulio Natta used a similar catalyst system to synthesize polymers with a stereoregular structure. This catalyst system was named the Ziegler–Natta catalyst. This discovery inaugurated the era of the mass production of polymers with stereoregular structures [7].

A typical Ziegler–Natta catalyst system comprises four essential components: TiCl_4_ acting as a catalyst precursor, MgCl_2_ as support, electron donors (Lewis bases), and alkyl aluminum as a catalyst activator. The primary function of the Ziegler–Natta catalyst lies in activating and controlling the olefin polymerization reaction. The transition metal compound acts as the active center of the catalyst, initiating the polymerization reaction and allowing the incorporation of the monomers. Meanwhile, the cocatalyst stabilizes the catalytic system and regulates the reaction rate [8,9].

Ziegler–Natta polymerization catalysts based on titanium (TiCl_4_/MgCl_2_) show high sensitivity to certain organic compounds that act as inhibitors. Some contaminants affect polymerization catalysts differently, depending on their degree of deactivation. For example, in the production of polypropylene (PP), the most harmful contaminants for catalytic deactivation include carbonyl sulfide (COS), carbon monoxide (CO), hydrogen sulfide (H_2_S), acetylene (C_2_H_2_), oxygen (O_2_) and arsine/phosphine. In linear low-density polyethylene (LLDPE) processes, the most concerning contaminants in the ethylene monomer feed stream are CO, O_2_, H_2_S, acetylene, and CO_2_ [10,11,12,13,14].

This research focuses on four types of organic compounds: methyl mercaptan, ethyl mercaptan, propyl mercaptan, and butyl mercaptan, which contain sulfur in their structure. They act as inhibitors of the Ziegler–Natta catalyst in the production of polypropylene. This study is not intended to carry out a detailed computational study. Therefore, molecular simulations based on density functional theory (DFT) studies were implemented in a complementary manner to explore the interactions between the molecules of these inhibitors and TiCl_4_, focusing on its influence on the catalytic activity, the melt flow rate, the molecular weight (Mw), and the production per metric ton of PP. This helped to complement and corroborate the results obtained with the experimental tests. Although numerous computational studies have focused on aspects such as the formation of the first active site of Ti, chain growth, the scaffold, its interaction with electron donors, and the interactions between different classes of molecule inhibitors and the active Ti center, no specific experimental and computational investigations have been carried out on these proposed compounds. This lack of prior research makes this study particularly innovative.

## 2. Materials and Methods

### 2.1. Standards and Reagents

Four types of mercaptans were used (methyl mercaptan, ethyl mercaptan, propyl mercaptan, and butyl mercaptan) provided by Merk in Darmstadt, Germany, and with a purity of 99.98%. For PP production, polymer-grade propylene (Shazand Petrochemical, Arak, Iran) was used, along with a fourth-generation Ziegler–Natta catalyst with MgCl_2_ support and, as an internal donor, diisobutyl phthalate (DIBP) supplied by Sud Chemie, Munich, Germany; The activator used was triethyl aluminum (TEA) of 99.97% purity from Merk, Darmstadt, Germany, diluted in n-heptane provided by Tosoh Finechem Corporation, Shiba, Tokyo. Additionally, tri-n-heptane and acetone were used. Another reagent was cyclohexylmethyldimethoxysilane (CMDMS) from Merk, Germany, used as an external donor, along with hydrogen and nitrogen.

### 2.2. Polymerization

**Figure 1:**
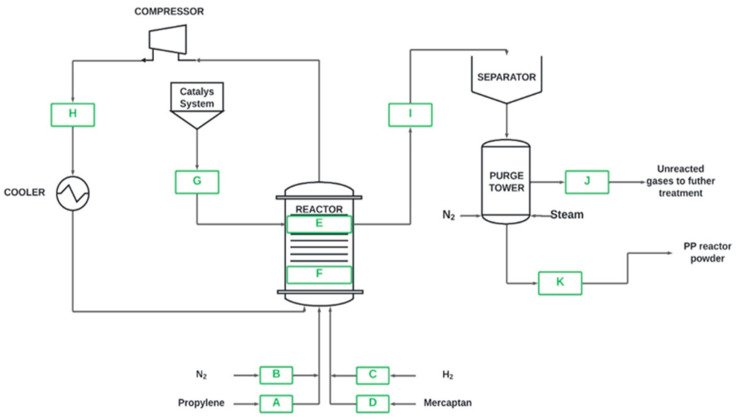
Production process diagram for polypropylene (PP).

Figure 1 corresponds to the PP polymerization scheme. Capital letters are assigned to each line of the process. Propylene (A), nitrogen (B), hydrogen (C), and mercaptan (D) are involved in the process. Gases generated during the reaction (E) come from the catalyst before (F) and inside (G) the reactor. Non-propylene that remains unreacted (H) is recycled in the process. The degassing stage of the PP resin (I) involves gases entrained (J) by nitrogen and water vapor, leading to the final resulting PP (K), as detailed in Table 1.

Once the polymerization was completed, acetone was added to stop the process, and subsequently, the suspension was transferred to a receiving flask maintained under a nitrogen (N_2_) atmosphere. The synthesized powder was washed thrice with 200 mL of heptane and then dried under vacuum at room temperature. The resulting polymer was stored under darkness, nitrogen, and controlled temperature conditions. Crucially, all steps of the procedure were meticulously carried out in a nitrogen atmosphere to avoid exposure to air.

The standard polymerization conditions were as follows: polymerization temperature of 72 °C, catalyst amount of 5.1 kg/h, triethylaluminium activator (TEAL), activator concentration of 0.26 kg/h, 30.1 g/h of H_2_ and 1.3 TM/ h of propylene at a pressure of 28 bar.

To ensure mercaptan concentrations in the propylene line, an Agilent Technologies 7890B GC-MS was used. The PP resin samples were analyzed using the Agilent 7694E headspace sampler, with a cycle time of 60 min and an oven set to 150 °C, according to Hernández’s method [15].

### 2.3. Melt Flow Index (MFI) and Average Molecular Weight (Mw)

The melt flow index (MFI) was determined using a Tinius Olsen MP1200 plastome, Horsham, PA, USA. The apparatus’s cylinder was at an operating temperature of 232 °C, and a 2.3 kg piston was used to displace the molten material. With the MFI data obtained, the Bremner method was applied to evaluate the average molecular weight of each polypropylene (PP) sample.

### 2.4. Infrared (IR) Spectroscopy

Infrared (IR) spectroscopic studies were conducted using a Nicolet iN10MX spectro-scope from Thermo Fisher Scientific (Thermo Scientific, Waltham, MA, USA) equipped with an iN10Z unit. Attenuated total reflection (ATR) mode was used. Spectra were recorded with a resolution of 4 cm^−1^, spanning a range from 400 to 4000 cm^−1^, allowing sensitive and accurate identification of various absorption bands.

### 2.5. Molecular Electrostatic Potentials

For this research, optimized geometry calculations for the various proposed inhibitors were carried out using Gaussian 16 Rev.A03 software with the B3LYP basis set, which has been recognized for its effectiveness in determining molecular structures. Electron densities and electrostatic potentials were then calculated using the 6-311G(d,p) basis set. The proposed molecules were surrounded by a three-dimensional surface that revealed the contour of constant electron density. On this surface, molecular electrostatic potentials were calculated and plotted. These potentials can be visualized with different levels of detail, but our current qualitative goal is to identify the sites most susceptible to nucleophilic, electrophilic, or free radical attacks.

### 2.6. Fukui Function

UKA FOKUI 2.00 software was used to obtain the quantitative values of the chemical descriptors and the local reactivity properties. These calculations offer detailed information on the local characteristics and reactivity of the inhibitors, the active site of the ZN catalyst, and its support, providing a deeper understanding of its behavior and chemical properties.

**Theoretical Complement of the Fukui Function**

Assessment of the Global and Local Reactivity Descriptors Methyl Mercaptan, Ethyl Mer-captan, Propyl Mercaptan and Butyl Mercaptan

The most effective method for studying the local selectivity of an inhibitor is through the condensed Fukui function. This function provides insight into how the electron densi-ty within the system changes. Mathematically, Fukui functions are derived from partial derivatives that relate the electron density to the number of electrons at a particular loca-tion within the molecule. These derivatives enable the quantification of that location’s ability to donate or accept electrons through nucleophilicity indices f+ (r), electrophilicity f-(r), and radical f 0 (r), where *qN* + 1, *qN* − 1, and *qN* are the electronic population of atom k in anionic, cationic, and neutral systems, respectively [16].

*fk*+ = *qN* + 1 − *qN*

*fk*− = *qN* − *qN* − 1

## 3. Results

During the chain propagation step in polypropylene (PP) synthesis, the transfer of propylene to Ti-PP occurs, where the olefin is incorporated into the PP-alkyl chain. This phase is sensitive to contaminants that act as inhibitors of various polarities and interact with the active Ti center, being influenced by different factors. In the case of the proposed mercaptans, a preference for coordination with the Ti center on the active Ti-MgCl_2_ surface is observed. This dynamic shows how the thiol group present in the mercaptans competes with the cocatalyst (TEAL) to bind to the Ti active site of –TiCl_3_, affecting the bonding of the olefin in the formation of propylene complexes and their insertion, therefore inhibiting the catalytic activity of the ZN system [17].

Initially, the sulfur in the structure coordinates the mercaptans with the Ti of the TiCl_4_/MgCl_2_ complex due to the predominance of the two free electron pairs of sulfur over those of propylene, which interact with Ti. The S-Ti interaction prevails over the formation of Ti-propylene complexes since the latter presents fewer barriers and a lower energy gain. To mitigate these interactions, it is essential to eliminate the impurities present in the system and resume the polymerization process.

Points I, J, and K were analyzed when detecting mercaptans at a trace level in the degassing process of the PP resins, as shown in Figure 1. This analysis gave important quantitative results, which were used to support the study on the inhibitory capacity of the samples of the different mercaptans reflected in Table 1.

The efficiency of the Ziegler–Natta catalyst is considerably affected by to the presence of inhibitors, which are adsorbed on the active surface of the TiCl_4_ crystalline network of the catalyst, favoring the loss of productivity. This interaction brings with it a lack of productivity due to an interference in the ability of the co-catalyst (TEAL) to compete for the active sites of the catalyst since it is occupied by the inhibitor that partially deactivates the catalyst, decreasing its catalytic activity, as reported in previous research by Hernandez-Fernandez [18,19].

### 3.1. Analysis of the Impact of Different Mercaptans on the Reduction of Metric Tons of Polypropylene (PP) Produced Depending on the Concentration at Various Sampling Points

The loss of productivity of the Ziegler–Natta catalytic system during the manufacture of PP or copolymers is directly related to the intervention of interactions that generate impurities with the Ti active center, generating a loss in the production of the desired polymer [20,21].

**Figure 2:**
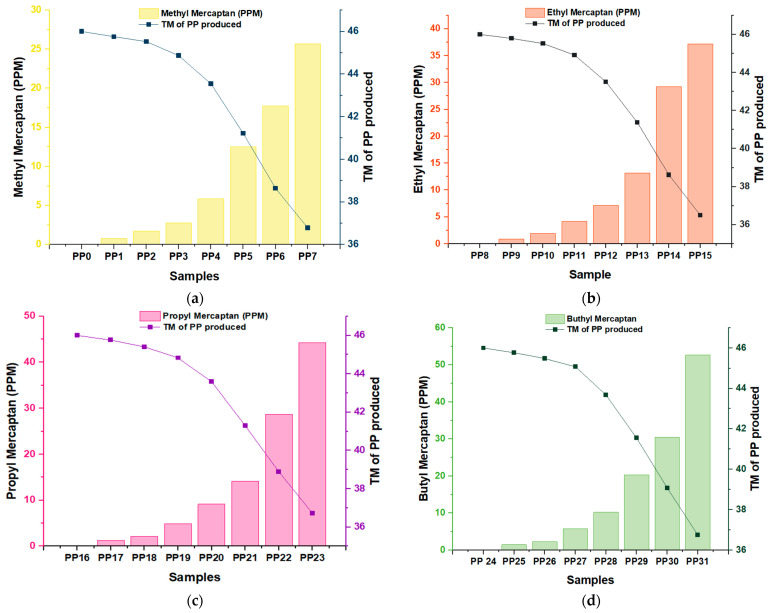
There is a loss of productivity per metric ton depending on the inhibitor (ppm), Methyl Mercaptan (**a**), Ethyl Mercaptan (**b**), Propyl Mercaptan (**c**), Butyl Mercaptan (**d**), and the type of sample.

The impact of various mercaptans on the reduction of catalytic activity was analyzed by considering multiple types of specific samples. The graphs in Figure 2 provide a detailed representation of how this decrease in production efficiency per metric ton of PP manifests as a function of the concentration of the inhibitors in each type of sample (PPn). In the first sample of PP0 (Graph a), PP8 (Graph b), PP16 (Graph c), and PP24 (Graph d) without any mercaptan (Figure 2), a catalyst productivity of 46 MT/kg was obtained in each graph. This indicates that the Ziegler–Natta catalyst worked well without adding mercaptan and achieved high polypropylene production. In sample PP1, where approximately 0.78 ppm of methyl mercaptan was added (Figure 2a), there was a production of 45.75 MT/kg, showing a production loss of 0.25 MT/kg; on the other hand, the ethyl mercaptan in sample PP8 with a concentration of 0.87 ppm (Figure 2b) had a production of PP of 45.79 TM/kg, and with this a production loss of 0.21 TM/kg was seen with a ppm concentration of ethyl mercaptan 0.09 times greater than the supplied concentration of methyl mercaptan. However, the production loss of ethyl mercaptan was lower than the production loss of methyl mercaptan. To confirm this trend, we analyzed the graph of another proposed mercaptan and compared it with graph (a). For this, propyl mercaptan was used (Figure 2c) because the concentration in ppm (1.23 TM/kg of propyl mercaptan) is 0.36 times greater than ethyl mercaptan and 0.45 times greater than the concentration supplied. Of methyl mercaptan, however, it can be seen that the production loss of propyl mercaptan was equal to the production loss of methyl mercaptan; despite the difference in concentration mentioned above, this same trend continues in the graph of butyl mercaptan (Figure 2d). This shows that methyl mercaptan acts as an inhibitor with a lower ppm concentration than the rest of the proposed mercaptans.

This is due to the difference in the molecular size of methyl mercaptan concerning the other inhibitors; by coordinating the sulfur with the titanium (S-Ti) on the active surface of the catalyst, the size of the chain allows the mercaptan to adsorb and couple correctly. This is a better way to achieve more effective inhibition. When analyzing all the graphs together, we see that, in each PP sample, when the molecular size of the inhibitory species increased, a higher concentration of butyl mercaptan, propyl mercaptan, and ethyl mercaptan was needed (just in this order, from highest to lowest concentration) to assimilate or equalize the production loss per metric ton of methyl mercaptan.

**Figure 3:**
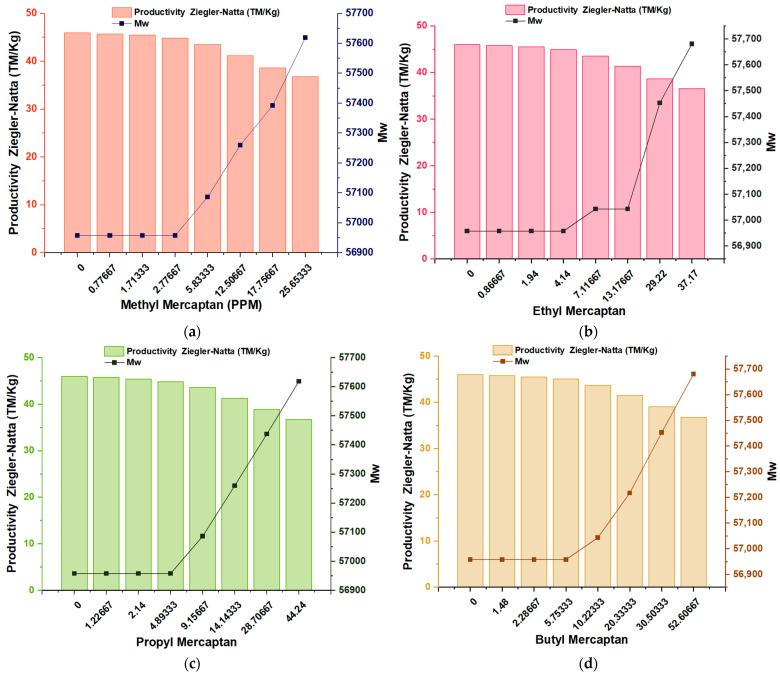
Effect of traces of Methyl Mercaptan (**a**), Ethyl Mercaptan (**b**), Propyl Mercaptan (**c**), and Butyl Mercaptan (**d**); Zigler–Natta productivity (MT/kg) about the Mw of the polymer.

The evaluation of the change in the average molecular weight (Mw) of the samples studied using the Bramner equation shows that in (Figure 3a–d), an increase in Mw was noted as the ppm concentration of the samples increased. In four different inhibitors, it can be seen that the value of Mw was 56,950 kDalton in the first four concentrations of each mercaptan; from this point on, the value of Mw increased constantly until reaching a value close to 57,700 kDalton.

This phenomenon indicates that these four types of mercaptans: methyl mercaptan, ethyl mercaptan, propyl mercaptan, and butyl mercaptan, exert a substantial impact on the properties of the material by forming stable complexes during the polymerization reaction. This influences the structure of the material, the polymer chain, and, therefore, its final properties, such as its average molecular mass (Mw).

### 3.2. Impact and Effects on Flow Index (MFI) and Mw of PP

**Figure 4:**
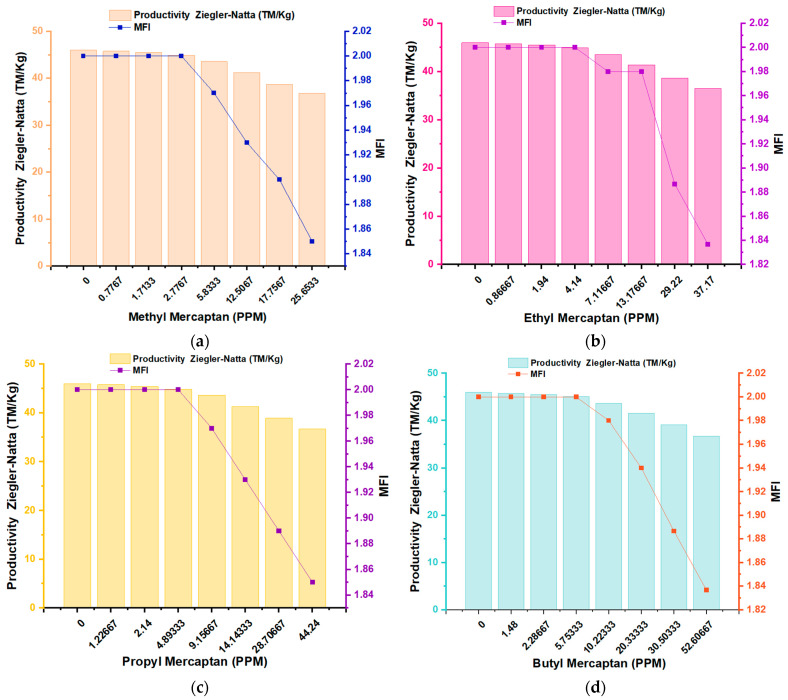
Effect of traces of Methyl Mercaptan (**a**), Ethyl Mercaptan (**b**), Propyl Mercaptan (**c**) and Butyl Mercaptan (**d**); Zigler–Natta productivity (MT/kg) over melt flow index (MFI).

In the analysis of the graphs of the different mercaptans regarding the MFI variable (Figure 4), it can be seen that the highest MFI was for a concentration of 0 ppm of the inhibitors; on the contrary, when the inhibitors reached their highest concentrations of (25.65 ppm) for methyl mercaptan, (37.17 ppm) for ethyl mercaptan, (44.24 ppm) for propyl mercaptan and (52.60 ppm) for butyl mercaptan, there was a decrease in the MFI for all samples, where it can be seen that the MFI value remained constant at 2.0 g/10 min in the first four concentrations of the different mercaptans. From this, we know how the MFI value decreases depending on the ppm concentration. In particular, it can be seen in graph (b), which corresponds to ethyl mercaptan, how after lowering the MFI value, it remains constant in two different concentrations, this being the value of 1.89 g/10 min; this reveals the effect of inhibitors on properties such as the melt index of the polymer. In addition, we can estimate the processing of the melt since the higher the MFI values, the greater the fluidity of the melt, and the lower the MFI values, the lower the fluidity of the polymer melt [22,23]. The MFI measurements in each copolymer sample were carried out in triplicate for each concentration of the mercaptans present.

**Figure 5:**
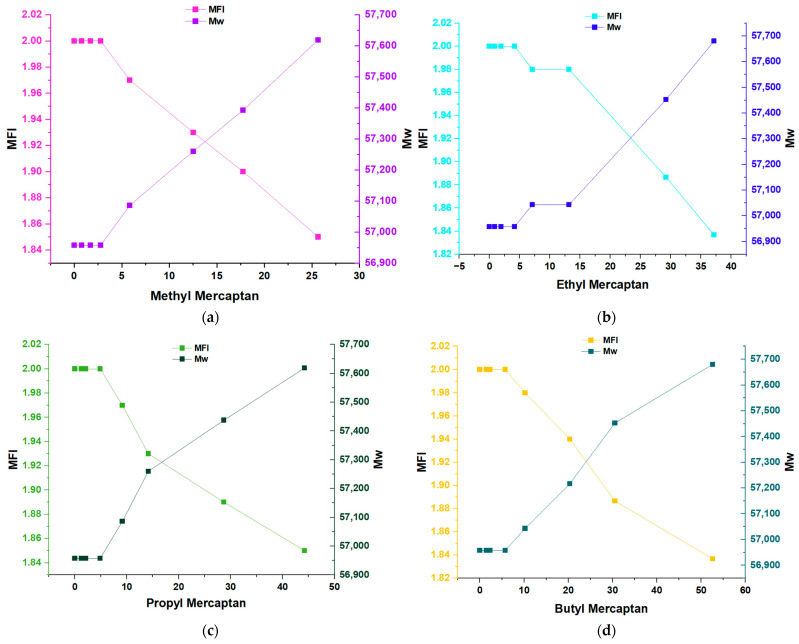
Flow rate and Mw of Methyl Mercaptan (**a**), Ethyl Mercaptan (**b**), Propyl Mercaptan (**c**) and Butyl Mercaptan (**d**)**.**

The Bramner equation shows the relationship between the MFI and the average molecular weight (Mw). In Figure 5a–d, an inversely proportional relationship is observed between the MFI and the Mw. Samples with an MFI of 2.0 g/10 min appeared with a Mw that was around 56,950 kDalton. The MFI between 1.97 and 1.93 g/10 min showed a Mw between 57,500 and 57,340 kDaltons. The samples of the copolymers with the highest MFI values ranged between 25.7 and 27.1 g/10 min, with a Mw that ranged between 35,304 and 34,854 kDaltons. It is important to highlight that an increase in molecular weight can negatively affect the processability of the polymer. Polymers with high molecular weight often exhibit higher viscosities, which complicates their handling during processes such as extrusion or injection molding. In applications that require greater flexibility or ease of processing, excessive molecular weight could be counterproductive.

### 3.3. Molecular Electrostatic Potential

The molecular electrostatic potential (MEP) map is a crucial tool for studying molecular structure and global reactivity, providing a detailed representation of a molecule’s charge distribution and electron availability. This method uses colors to outline the different regions of the molecule based on their electron density [24,25]. In the MEP, red areas indicate a higher electron density, suggesting the presence of nucleophilic sites in the molecule, that is, regions with a high probability of donating electrons. In contrast, the blue areas represent an electron deficiency, signaling the presence of electrophilic sites, where the molecule has a greater affinity for accepting electrons.

These characteristics are essential to understanding the chemical reactivity of the molecule since they allow us to predict how it will interact with other chemical species. By identifying the nucleophilic and electrophilic sites in the MEP, it is possible to determine which areas of the molecule are most likely to participate in chemical reactions and how they may interact with other substances.

**Figure 6:**
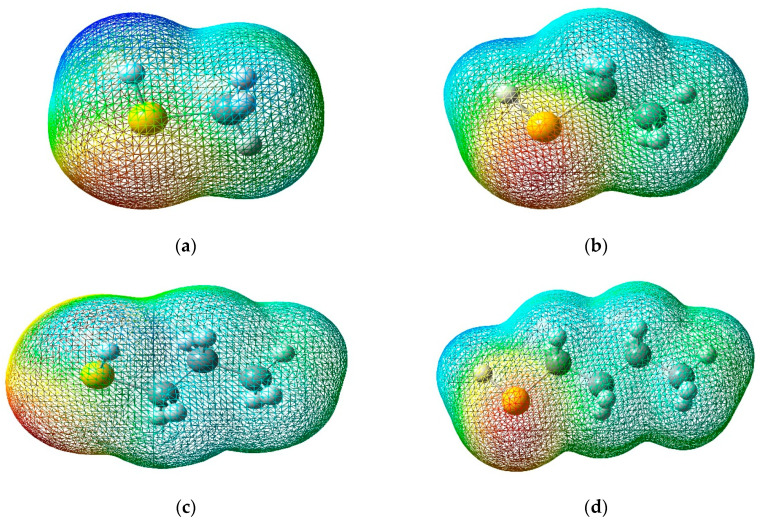
Molecular electrostatic potential map; (**a**) Methyl Mercaptan (**b**) Ethyl Mercaptan (**c**) Propyl Mercaptan (**d**) Butyl Mercaptan.

Figure 6 shows a three-dimensional representation of the electrostatic effect, covering a range of values that goes from −3.907 × 10^−2^ to 3.907 × 10^−2^ for methyl mercaptan, −2.854 × 10^−2^ up to 2.854 × 10^−2^ for ethyl mercaptan, −2.867 × 10^−2^ to 2.867 × 10^−2^ for propyl mercaptan and −2.885 × 10^−2^ and 2.885 × 10^−2^ for butyl mercaptan.

According to Figure 6, for these four types of mercaptans, the blue, green, and red colors represent the regions with the most positive electrostatic potential, zero potential, and the most negative electrostatic potential, respectively. The red and yellow regions are mainly found on the sulfur atom, indicating that it is the most reactive site for an electrophilic attack. On the other hand, the blue regions around the hydrogen atoms are the most reactive sites for a nucleophilic attack.

**Figure 7:**
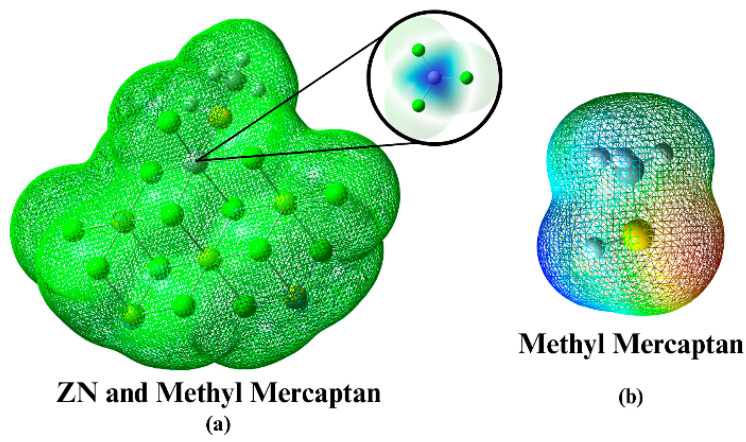
(**a**) Electrostatic potential map of the Ziegler–Natta catalyst; (**b**) Electrostatic potential map of Methyl Mercaptan.

This MEP information is relevant to the ZN catalyst inhibition approach because it suggests that the proposed mercaptans can act as inhibitors by interacting with the active sites on the catalyst. The central region of the Ziegler–Natta catalyst stands out for its blue tone, located on the titanium atom. The blue tone shown in Figure 7 suggests a more significant electron deficiency in that area and, therefore, a greater tendency to accept electrons.

This information provided by the MEP indicates that the proposed mercaptans could act as inhibitors of the ZN catalyst. This is caused by the interactions with the catalyst in its electrophilic regions. Thus, they can form a stable complex and consequently negate the interactions. The catalyst may have the desired reagent (be it AlEt_3_ or the same PP), preventing the catalytic process from being carried out correctly [15,16,26,27,28,29,30,31,32,33,34,35,36,37,38,39,40,41,42,43,44,45,46,47,48,49,50].

### 3.4. Analysis of Methyl Mercaptan, Ethyl Mercaptan, Propyl Mercaptan, and Butyl Mercaptan as Inhibitors of the ZN Catalyst

This study focuses on studying the trends of mercaptans as inhibitors of the ZN catalyst using density functional theory (DFT) at the B3LYP/6-311G(d,p) level. The Fukui index was used to investigate local reactivity to predict the most likely sites where nucleophilic and electrophilic attacks may occur. The repercussions that a compound with the ability to inhibit and thus reduce catalytic activity can have are often due to how the inhibitor molecule binds to the surface of the metal. This interaction can occur in two ways: physical (physisorption) or chemical (chemisorption); this depends directly on the strength of this connection. For chemisorption, one of the reactive molecules acts as an electron pair donor; on the other hand, a different molecule acts as an electron pair acceptor [30,31,32].

#### Fukui Features

To understand the Fukui function, one must know that it is divided into two parts: there is the nucleophilic Fukui function, ƒr^+^, which indicates the areas with a greater probability of suffering a nucleophilic attack by electron-rich species, and in contrast to this there is the electrophilic Fukui function, ƒr^−^, which shows the areas where there is a greater probability of an electrophilic attack by a species lacking electrons. In this way, the Fukui function helps in understanding and identifying the sites most likely to react in a molecule. The dual descriptor Δfr is used, understood as the difference between ƒr^+^ and ƒr^−^. If Δf has a positive character, it indicates a greater probability of being attacked by a nucleophilic species in those areas; in contrast to this, if Δfr is obtained with a negative character, this indicates a greater probability of being attacked by an electrophilic species. To have a greater understanding of the reactive nature of the molecule, calculations and tables were carried out for the quantitative interpretation of the Fukui functions (ƒ^0^, ƒ^+^ and ƒ^−^) for each site of the molecule. The information it provides helps to interpret the qualitative reactivity and, with this, the selectivity of specific sites within the molecule. Table 2, Table 3, Table 4 and Table 5 show values associated with the Fukui functions of each type of mercaptan, thereby identifying which areas of the molecule are most likely to react and how this can happen—a reaction with different chemical species [33,34].

**Figure 8:**
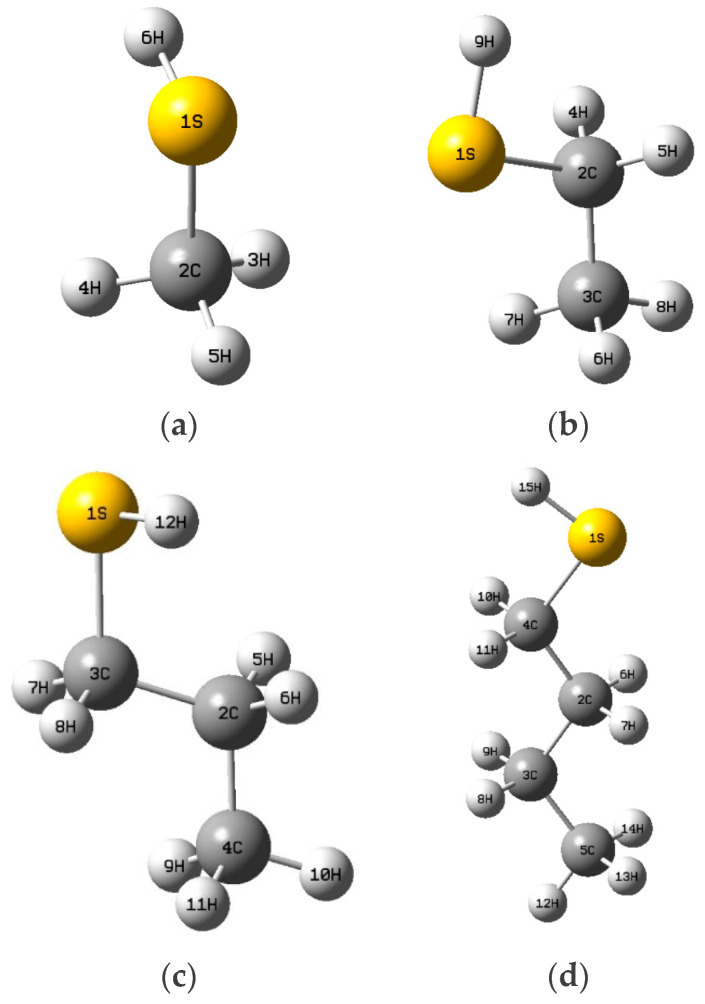
Spatial Conformation of Methyl Mercaptan (**a**), Ethyl Mercaptan (**b**), Propyl Mercaptan (**c**), Butyl Mercaptan (**d**).

Calculations of the Fukui function for each mercaptan have allowed the identification of the sites most likely to undergo nucleophilic attack by the mercaptans (see Table 1, Table 2, Table 3, Table 4 and Table 5). Higher *f*^−^ values are obtained at atom number 1, representing sulfur (see Figure 8), with values ranging from *f*^−^ (0.9095–0.7416), also highlighting its susceptibility to electrophilic attacks. In contrast, for methyl mercaptan, the 2C carbon atom is more prone to nucleophilic attacks with an *f*^+^ value (0.6773) compared to other atoms. For ethyl mercaptan, the trend of the carbon atom prevails with an *f*^+^ value (0.2927), and the 3C carbon atom shows a more notable susceptibility with an *f*^+^ value (0.3019). On the other hand, for propyl mercaptan and butyl mercaptan, the 2C carbon atom changes its trend, drastically decreasing the probability of a nucleophilic attack; however, the electrophilic trend increases significantly with an *f*^+^ value (0.3692) for the 3C carbon atom, and additionally, the 4C carbon atom increases with an *f*^+^ value (0.3444). For butyl mercaptan, the 3C carbon atom decreases drastically for *f*^+^ (0.1348) and slightly for the 4C carbon atom *f*^+^ (0.3328).

A greater reactivity and probability of electrophilic attacks are interpreted in the case of sulfur atoms (represented by the number 1S). Likewise, the quantitative values show that sulfur is the atom most prone to radioactive attacks. This can be evidenced since they present Δf (−0.866) for methyl mercaptan, Δf (−0.84) for ethyl mercaptan, Δf (−0.7994) for propyl mercaptan, and Δf (−0.6933) for butyl mercaptan.

The results in Table 6 reveal that the chlorine atoms in the two, three, and four positions of TiCl_4_ are particularly susceptible to electrophilic attacks. In contrast, the titanium atom at position one is the primary site for a nucleophilic attack.

**Figure 9:**
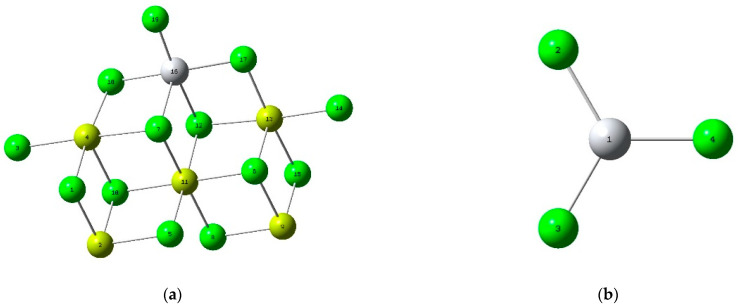
Spatial conformation of the Ziegler–Natta catalyst (**a**) and its active site (**b**).

Furthermore, it was observed that both the titanium in position one and the chlorine atoms in positions two, three, and four are the most vulnerable to free radical attacks (see Figure 9).

Regarding the support spatial conformation of the Ziegler–Natta catalyst (a) and its active site (b). e (Table 7), it has been identified that the atoms in positions two and nine are particularly susceptible to nucleophilic attacks. These atoms are of particular interest since, according to previous research, alcohols react with magnesium cations in these positions. To support these findings, data have been compiled in Table 7, which presents the specific values of *f*^−^, *f*^+^, *f*^0^, Δf for each of the atoms on the catalyst’s surface. These values provide a solid basis for understanding the tendency of the atoms above to be affected by nucleophilic attacks.

### 3.5. Experimental Analysis by FTIR of the Reaction Product of ZN With Each Mercaptan of Interest

Analyzing the Fourier transform infrared (FTIR) spectra of the ZN catalyst samples after their reactions with each of the four mercaptans under study in this experiment offers a detailed view of their interactions with the active center of titanium. FTIR spectra reveal significant changes in the mercaptans’ structure and chemical bonds when they come into contact with the catalyst, providing a deeper understanding of the catalysis processes and the surface dynamics in Ziegler–Natta systems. Figure 7 shows five spectra identified as ZN–methyl mercaptan, ZN–ethyl mercaptan, ZN–propyl mercaptan, ZN-butyl mercaptan, and ZN. The Ti–Cl bond is detected in the region from 618 to 555 cm^−1^ for the ZN complex [37]. This indicates the formation of complexes or the adsorption of species on the catalytic surface, where the Ti–Cl bond is identified in the range of 603 to 617 cm^−1^, showing a closeness in the region of this bond.

In each spectrum, a peak can also be seen at 1510 cm^−1^, which is characteristic of the Cl–Mg bond typical of the catalyst support. Peaks are observed between 1474 cm^−1^ and 1510 cm^−1^, corresponding to the stretching vibration of the C–H bonds, particularly associated with the -CH_2_ group. Between 2972 and 2990 cm^−1^, peaks typical of -CH_3_ are identified. These alkyl groups are present in the aliphatic chain of the four mercaptans investigated in this work. In the ZN spectra with each mercaptan, peaks are observed between 450 and 480 cm^−1^, which, compared with the literature, are characteristic of the vibration of the Ti–S bond. A shift of the peak to the left can also be observed for the Ti–Cl bond at 493 cm^−1^.

**Figure 10:**
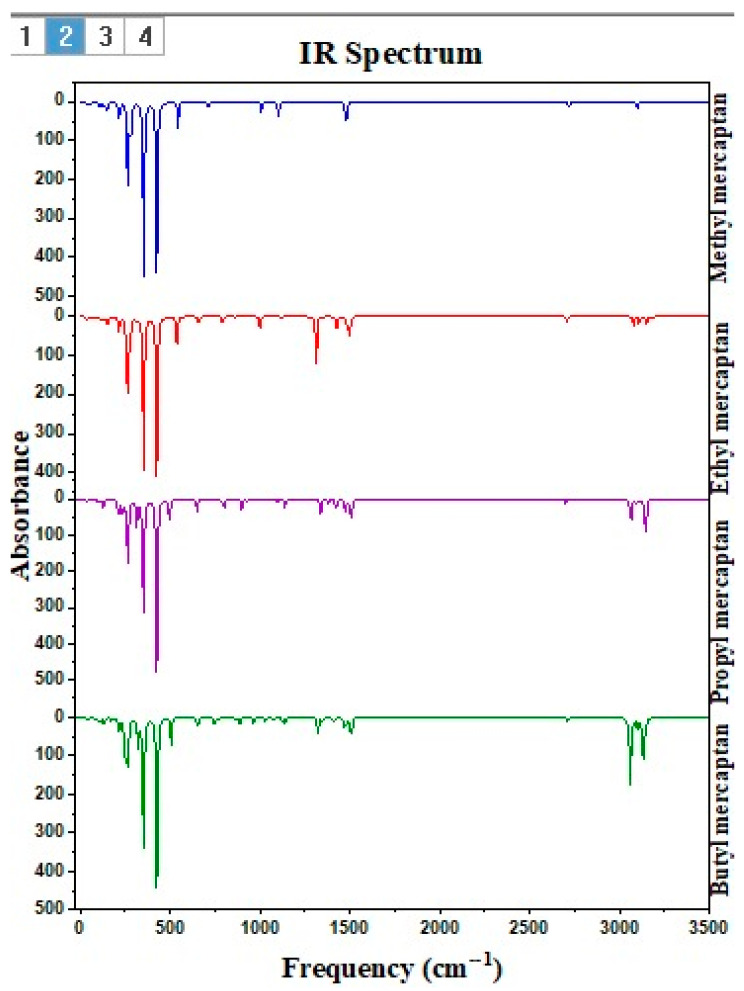
The infrared spectrum of ZN-methyl mercaptan, ZN-ethyl mercaptan, ZN-propyl mercaptan, and ZN-butyl mercaptan.

Figure 10 shows the possible torsion–rotation for a robust parallel band centered at 644 cm^−1^ and a weaker band centered at 727 cm^−1^ for the C–S bond in the Ti–S–C junction in the ZN–methyl mercaptan complex. Such bands in similar regions were also identified when FTIR analyzed pure methyl mercaptan [38,39].

Table 8 shows the frequencies identified in Figure 10 for the titanium–sulfur (Ti-S), titanium–chlorine (Ti–Cl), chlorine–magnesium (Cl–Mg), sulfur–carbon (S–CH_3_ and S–CH_2_) and carbon–hydrogen (CH_3_ and CH_2_) for the complexes ZN–methyl mercaptan, ZN–ethyl mercaptan, ZN–propyl mercaptan and ZN–butyl mercaptan. In the spectra corresponding to the mercaptans, specifically ZN–methyl mercaptan, ZN–ethyl mercaptan, ZN–propyl mercaptan, and ZN–butyl mercaptan, a peak of crucial importance was identified that confirms the reactions indicated in Table 9, corresponding to the Cl–Ti–S–CH_2_ bond and the Cl–Ti–S–CH_3_ bond in the literature. The values reported for this type of vibration correspond to 644 and 728 cm^−1^.

## 4. Discussion

This study of the presence of mercaptans in the Ziegler–Natta (ZN) catalyst was carried out to determine and evaluate the percentage of production loss measured in metric tons per kilogram of solvent (TM/kg) and, in addition, to analyze the effects of these compounds on the properties and final characteristics of polypropylene (PP). To measure the efficiency of the four proposed mercaptans, a comparison was made with a study previously carried out by Hernández Fernández on an evaluation of the reactivity of methanol and hydrogen sulfide waste with the catalyst [35]. Mercaptans are the direct focus in the inhibitory interaction of methanol since mercaptans are sulfur analogs of alcohols [36]. For this, it is necessary to delve into the mercaptans’ possible interactions with the ZN catalyst’s active site during the synthesis of PP to determine its inhibitory tendency. It can be seen in the results presented in Table 1 that the most potent inhibitory capacity belongs to methyl mercaptan since it produces a productivity loss of 0.54% with a concentration of only 0.78 ppm compared to butyl mercaptan, which has with the lowest inhibitory capacity among the four mercaptans with a loss of 0.51% but requiring a concentration of 1.48 ppm. When comparing these results with the methanol present in the study carried out by Hernández, the methanol does not manage to match the values of the productivity loss of the mercaptans, being a value of methanol 0.35% with a concentration of 4.3 ppm, evidencing a much higher concentration but with a lower productivity loss value. The computational study was carried out to support the experimental research following specific guidelines, such as the analysis of the chemical nature of the inhibitors. Previous research has identified other inhibitors that affect ZN productivity [15,16,40,41,42,43,44,45,46,47,48,49,50].

The UKA FOKUI 2.00 software was used to obtain the quantitative values of the mercaptans to determine the local reactivity properties (see Table 2, Table 3, Table 4 and Table 5), where the numerical values of the sulfur atoms found are shown. In position one (see Figure 8), it is observed that the Δfr for the sulfur atoms are Δf (−0.866) for methyl mercaptan, Δf (−0.84) for ethyl mercaptan, Δf (−0.7994) for propyl mercaptan, Δf (−0.6933) for butyl mercaptan, showing a nature susceptible to electrophilic attacks. Hernandez’s research shows the oxygen atom present in position five (see Figure 10) in the methanol molecule also presents this same tendency but with a lower Δfr (see Table 5), this being Δf (−0.280), evidencing a low susceptibility to electrophilic attacks compared to mercaptans. In the electrostatic potential (MEP) map, the values varied from −3.907 × 10^−2^ to 3.907 × 10^−2^ for methyl mercaptan, from −2.854 × 10^−2^ to 2.854 × 10^−2^ for ethyl mercaptan, from −2.867 × 10^−2^ to 2.867 × 10^−2^ for propyl mercaptan, and from −2.885 × 10^−2^ to 2.885 × 10^−2^ for butyl mercaptan, the sites most prone to electrophilic attack being those around the atom of sulfur. Similarly, the methanol molecule exhibits an electrostatic potential range varying from −6.288 × 10^−2^ to 6.288 × 10^−2^ eV. This shows that mercaptans exhibit a superior inhibitory effect at a quantitative and qualitative level. This research could lead to more in-depth studies on the inhibitory capacity of mercaptans in the polymerization process at an industrial level, which is a novel and unexplored area.

## 5. Conclusions

This research demonstrates that trace-level concentrations of four different mercaptans negatively affect the production of polypropylene (PP) and the catalytic activity of the Ziegler–Natta (ZN) catalyst. Increasing mercaptan concentrations inversely impacted the ZN catalyst’s productivity, with methyl mercaptan being the most potent inhibitor, reducing productivity with the smallest concentration. The study also identified an inverse relationship between the polymer’s melt index and molecular weight (Mw), while higher mercaptan concentrations correlated directly with increased Mw. These findings indicate that mercaptans significantly alter the polymer structure, potentially affecting its performance during manufacturing and in subsequent applications, primarily due to their strong tendency to donate electrons to the titanium active center of the ZN catalyst, thus acting as inhibitors in the polymerization process.

## Figures and Tables

**Table 1:** Amount of methyl mercaptan, ethyl mercaptan, propyl mercaptan, and butyl mercaptan introduced during the polymerization process. Measurement of Mw, MFI, % Zigler–Natta productivity, and % lost production in the catalytic system (Average table).

Methyl Mercaptan (PPM)	0.00	0.78	1.71	2.78	5.83	12.51	17.76	25.65
TM of PP Produced	46.00	45.75	45.52	44.87	43.54	41.22	38.64	36.78
Productivity Ziegler–Natta (TM/Kg)	46.00	45.75	45.52	44.87	43.54	41.22	38.64	36.78
% Productivity Loss	0.00	0.54	1.04	2.46	5.34	10.40	16.00	20.04
MFI	2.00	2.00	2.00	2.00	1.97	1.93	1.90	1.85
% MFI loss	0	0	0	0	1.50	3.50	5.0	7.50
Ethyl Mercaptan (PPM)	0.00	0.87	1.94	4.14	7.12	13.18	29.22	37.17
TM of PP Produced	46.00	45.79	45.52	44.91	43.51	41.36	38.62	36.50
Productivity Ziegler–Natta (TM/Kg)	46.00	45.79	45.52	44.91	43.51	41.36	38.62	36.50
% Productivity Loss	0.00	0.46	1.04	2.36	5.41	10.08	16.05	20.66
MFI	2.00	2.00	2.00	2.00	1.98	1.98	1.89	1.84
% MFI loss	0	0	0	0	1	1	5.65	8.15
Propyl Mercaptan (PPM)	0.00	1.23	2.14	4.89	9.16	14.14	28.71	44.24
TM of PP Produced	46.00	45.75	45.40	44.83	43.59	41.29	38.89	36.73
Productivity Ziegler–Natta (TM/Kg)	46.00	45.75	45.40	44.83	43.59	41.29	38.89	36.73
% Productivity Loss	0.00	0.54	1.30	2.55	5.23	10.24	15.46	20.16
MFI	2.00	2.00	2.00	2.00	1.97	1.93	1.89	1.85
% MFI loss	0	0	0	0	1.50	3.50	5.50	7.50
Butyl Mercaptan (PPM)	0.00	1.48	2.29	5.75	10.22	20.33	30.50	52.61
TM of PP Produced	46.00	45.76	45.48	45.08	43.68	41.55	39.08	36.75
Productivity Ziegler–Natta (TM/Kg)	46.00	45.76	45.48	45.08	43.68	41.55	39.08	36.75
% Productivity Loss	0.00	0.51	1.13	2.00	5.04	9.67	15.05	20.12
MFI	2.00	2.00	2.00	2.00	1.98	1.94	1.89	1.84
% MFI loss	0	0	0	1	1	3	6.67	8.16

**Table 2:** Local descriptors for Methyl Mercaptan.

#	*f* ^−^	*f* ^+^	*f* ^0^	Δf
1	0.9095	0.0435	0.4765	−0.866
2	0.0272	0.6773	0.3523	0.6501
3	0.0314	0.0676	0.0495	0.0362
4	0.0314	0.0675	0.0495	0.0361
5	0	0.0739	0.037	0.0739
6	0.0005	0.0701	0.0353	0.0696

**Table 3:** Local descriptors for Ethyl Mercaptan.

#	*f* ^−^	*f* ^+^	*f* ^0^	Δf
1	0.8866	0.0466	0.4666	−0.84
2	0.0201	0.2927	0.1564	0.2726
3	0.0045	0.3019	0.1532	0.2974
4	0.0352	0.074	0.0546	0.0388
5	0.0352	0.074	0.0546	0.0388
6	0.0092	0.0303	0.0198	0.0211
7	0.0092	0.0303	0.0198	0.0211
8	0	0.0506	0.0253	0.0506
9	0	0.0995	0.0497	0.0995

**Table 4:** Local descriptors for Propyl Mercaptan.

#	*f* ^−^	*f* ^+^	*f* ^0^	Δf
1	0.8271	0.0277	0.4274	−0.7994
2	0.0397	0.0337	0.0367	−0.006
3	0.0654	0.3692	0.2173	0.3038
4	0.0288	0.3444	0.1866	0.3156
5	0.0046	0.0145	0.0095	0.0099
6	0.001	0.0385	0.0197	0.0375
7	0.0012	0.037	0.0191	0.0358
8	0.0278	0.0334	0.0306	0.0056
9	0.0011	0.0132	0.0071	0.0121
10	0.0024	0.0296	0.016	0.0272
11	0.0006	0.0242	0.0124	0.0236
12	0.0004	0.0346	0.0175	0.0342

**Table 5:** Local descriptors for Butyl Mercaptan.

#	*f* ^−^	*f* ^+^	*f* ^0^	Δf
1	0.7416	0.0483	0.395	−0.6933
2	0.0491	0.0423	0.0457	−0.0068
3	0.0361	0.1348	0.0855	0.0987
4	0.0143	0.3328	0.1735	0.3185
5	0.0018	0.0999	0.0508	0.0981
6	0.0302	0.0171	0.0237	−0.0131
7	0.0313	0.014	0.0226	−0.0173
8	0.0154	0.0209	0.0182	0.0055
9	0.0111	0.0238	0.0174	0.0127
10	0.0344	0.0665	0.0504	0.0321
11	0.0348	0.0669	0.0509	0.0321
12	0	0.0117	0.0058	0.0117
13	0.0001	0.0121	0.0061	0.012
14	0	0.0109	0.0055	0.0109
15	−0.0001	0.0982	0.049	0.0983

**Table 6:** Fukui functions for TiCl_4_.

#	*f^−^*	*f* ^+^	*f* ^0^	Δf
1	0.0001	0.7968	0.3986	0.7968
2	0.3741	0.0506	0.2125	−0.3234
3	0.163	0.0503	0.1073	−0.1137
4	0.373	0.0513	0.2125	−0.3229
5	0.0878	0.0509	0.0693	−0.0368

**Table 7:** Fukui functions for ZN support.

#	*f^−^*	*f* ^+^	*f* ^0^	Δf
1	0.0313	0.0304	0.0309	−0.0009
2	0.0013	0.4433	0.2223	0.442
3	0.5554	0.0032	0.2793	−0.5522
4	0.0109	0.0029	0.0069	−0.008
5	0.0011	0.0351	0.0181	0.034
6	0.0004	0.0198	0.0101	0.0194
7	0.0003	0.0036	0.0019	0.0033
8	0.0007	0.0287	0.0147	0.028
9	0.0007	0.3607	0.1807	0.36
10	0.0005	0.0236	0.0120	0.0231
11	0.0003	0.0123	0.0063	0.012
12	0.0000	0.0037	0.0019	0.0037
13	0.0068	0.0017	0.0043	−0.0051
14	0.3523	0.0027	0.1775	−0.3496
15	0.0194	0.0248	0.0221	0.0054
16	0.0064	0.0014	0.0039	−0.005
17	0.0060	0.0006	0.0033	−0.0054
18	0.0060	0.0014	0.0037	−0.0046
19	0.0002	0.0001	0.0002	−0.0001

**Table 8:** Frequencies (cm^−1^) of characteristic bonds present in ZN–Mercaptans.

Bonds	ZN-Propyl	ZN-Methyl Mercaptan	ZN-Ethyl Mercaptan	ZN-Propyl Mercaptan	ZN-Butyl Mercaptan
Ti-S	--------	430	445	475	477
Ti-Cl	618–555	493	725	591	599
Cl-Mg	1510	1456	1510	1625	1634
Cl-Ti-S-CH_2_	--------	--------	644–720	647–725	642–728
Cl-Ti -S-CH_3_	--------	644–722	--------	--------	--------
-CH_3_	2995–2969	3058	3054	2997	3115–2986
-CH_2_	1517–1480	--------	1518–1444	1511–1449	1513–1467

**Table 9:** Symbolic representation of the reactions of the process.

Reagents		Products
TiCl_4_ + CH_3_S	→	CH_3_STiCl_4_
TiCl_4_ + CH_3_CH_2_S	→	CH_3_CH_2_S TiCl_4_
TiCl_4_ + CH_3_CH_2_CH_2_S	→	CH_3_CH_2_CH_2_STiCl_4_
TiCl_4_ + CH_3_CH_2_CH_2_CH_2_S	→	CH_3_CH_2_CH_2_CH_2_STiCl_4_

## Data Availability

The original contributions presented in the study are included in the article, further inquiries can be directed to the corresponding author.
